# CoCrMo‐Nanoparticles induced peri‐implant osteolysis by promoting osteoblast ferroptosis via regulating Nrf2‐ARE signalling pathway

**DOI:** 10.1111/cpr.13142

**Published:** 2021-10-11

**Authors:** Yiming Xu, Weilin Sang, Yiming Zhong, Song Xue, Mengkai Yang, Cong Wang, Haiming Lu, Renchun Huan, Xinjie Mao, Libo Zhu, Chuanglong He, Jinzhong Ma

**Affiliations:** ^1^ Department of Orthopedics Shanghai General Hospital, Shanghai Jiao Tong University School of Medicine Shanghai China; ^2^ Shanghai Bone Tumor Institution Shanghai China; ^3^ State Key Laboratory for Modification of Chemical Fibers and Polymer Materials, College of Chemistry, Chemical Engineering and Biotechnology Donghua University Shanghai China

## Abstract

**Objectives:**

Aseptic loosening (AL) is the most common reason of total hip arthroplasty (THA) failure and revision surgery. Osteolysis, caused by wear particles released from implant surfaces, has a vital role in AL. Although previous studies suggest that wear particles always lead to osteoblast programmed death in the process of AL, the specific mechanism remains incompletely understood and osteoblast ferroptosis maybe a new mechanism of AL.

**Materials and Methods:**

CoCrMo nanoparticles (CoNPs) were prepared to investigate the influence of ferroptosis in osteoblasts and calvaria resorption animal models. Periprosthetic osteolytic bone tissue was collected from patients who underwent AL after THA to verify osteoblast ferroptosis.

**Results:**

Our study demonstrated that CoNPs induced significant ferroptosis in osteoblasts and particles induced osteolysis (PIO) animal models. Blocking ferroptosis with specific inhibitor Ferrostatin‐1 dramatically reduced particle‐induced ferroptosis in vitro. Moreover, in osteoblasts, CoNPs significantly downregulated the expression of Nrf2 (nuclear factor erythroid 2‐related factor 2), a core element in the antioxidant response. The overexpression of Nrf2 by siKeap1 or Nrf2 activator Oltipraz obviously upregulated antioxidant response elements (AREs) and suppressed ferroptosis in osteoblasts. Furthermore, in PIO animal models, the combined utilization of Ferrostatin‐1 and Oltipraz dramatically ameliorated ferroptosis and the severity of osteolysis.

**Conclusions:**

These results indicate that CoNPs promote osteoblast ferroptosis by regulating the Nrf2‐ARE signalling pathway, which suggests a new mechanism underlying PIO and represents a potential therapeutic approach for AL.

## Graphical abstract

CoCrMo‐Nanoparticles induced peri‐implant osteolysis by promoting osteoblast ferroptosis via regulating Nrf2‐ARE signalling pathway. Blocking ferroptosis with Ferrostatin‐1 and Oltipraz significantly ameliorated osteolysis induced by implanted particles, which suggests a new mechanism underlying particle‐induced osteolysis and represents a potential therapeutic approach for aseptic loosening.

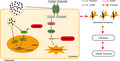

## 1 INTRODUCTION

Total hip arthroplasty (THA) serves as one of the most successful orthopaedic surgeries that are widely used for the treatment of severe degenerative, post‐traumatic, and other end‐stage diseases of the hip joint.^1^ Nevertheless, periprosthetic aseptic loosening and subsequent osteolysis always result in THA failure and surgical revision.^2^, ^3^ The main cause of revision surgeries is due to wear particles, which are created from implanted prostheses.^4^ Wear particles can lead to variable adverse local tissue responses, which disrupt the homeostasis between bone formation and resorption. Many different types of cells, including osteoclasts, osteoblasts, macrophages, fibroblasts, and lymphocytes, contribute to the occurrence of osteolysis to varying degrees.^5^, ^6^ Numerous researches have focused on osteoclasts and macrophages, which are mainly related to increased bone resorption during osteolysis.^7^ Among the cell lines stimulated by wear particles, osteoblasts are one of the earliest cells exposed to wear particles; however, the influence of wear particles on osteoblasts has not attracted enough attention.^8^ Osteoblasts are of crucial importance in the secretion of extracellular matrix (mainly type I collagen) in vivo, which are the exclusive cells engaged with bone formation.^9^, ^10^ Wear particles have been shown to have adverse effects on the proliferation and survival of osteoblasts.^11^ The mechanism underlying the interaction between wear particles and osteoblasts remains unclear.

Ferroptosis, a novel form of regulated cell death pathway, is characterized by the accumulation of lethal lipid reactive oxygen species (ROS).^12^, ^13^ It is inconsistent with the classical apoptotic cell death programmes, including apoptosis, necroptosis, and other classical non‐apoptotic cell death programmes, in terms of morphological, biochemical, and genetic studies.^14^ Current research has proved that ferroptosis can be induced by the blockade of cystine/glutamate system Xc^−^ activity, downregulation of glutathione peroxidase 4 (GPX4), and an increase in lipid ROS.^15^, ^16^ System Xc^−^ (composed of SLC7A11, solute carrier family 7, and SLC3A2, solute carrier family 3 member 2) is a transmembrane cysteine‐glutamate antiporter that specifically transfers extracellular cystine to intracellular glutamate as the origin of glutathione (GSH), which is the major cellular antioxidant.^17^ GPX4, another core regulator of ferroptosis, reduces organic hydroperoxides and lipid peroxides through the consumption of GSH.^18^, ^19^, ^20^ The inhibition of GPX4 and System Xc^−^ can result in an uncontrolled increase in lipid peroxidation, resulting in ferroptosis.^21^ Moreover, acyl‐CoA synthetase long‐chain family member 4 (ACSL4), a vital factor in metabolism‐associated diseases, has been linked to the promotion of the esterification of arachidonoyl (AA) and adrenoyl into phosphatidylethanolamine (PE), which is strongly associated with ferroptosis and critically determines the sensitivity of the cells to ferroptosis.^22^, ^23^ Many diseases have been reported to be related to ferroptosis, including Alzheimer's disease,^24^ carcinogenesis,^25^ intracerebral haemorrhage,^26^ traumatic brain injury,^27^ stroke,^28^ and ischaemia‐reperfusion injury.^22^ However, the relationship between ferroptosis and particles induced osteolysis (PIO) is still unknown.

Nrf2, the nuclear factor erythroid 2‐related factor 2, is of crucial significance to the antioxidant response that protects cells from different kinds of cell death‐induced oxidative stress, including ferroptosis.^29^, ^30^ Nrf2 binds its promoter to target genes to regulate cellular redox homeostasis,^17^, ^31^ which contains antioxidant response elements (AREs), such as heme oxygenase‐1 (HO‐1) and quinone oxidoreductase‐1 (NQO‐1). Numerous studies have shown that Nrf2 can be continually degraded by Kelch‐like ECH‐associated protein 1 (Keap1), and the suppression of Keap1 activates the Nrf2‐ARE pathway under various oxidative stress conditions.^29^, ^32^ The ROS content around the prosthesis increases significantly during wear‐particle‐mediated osteolysis.^33^, ^34^ However, how Nrf2 regulates the process of wear‐particle‐mediated osteolysis requires further study.

In the present study, we hypothesized osteoblast ferroptosis was an important reason for PIO. We used the most common clinically CoCrMo Nanoparticles (CoNPs) as wear particles for further research. Our study demonstrated that CoNPs induced significant osteoblast ferroptosis both in AL clinical samples, osteoblasts, and calvaria resorption animal models. Ferroptosis‐specific inhibitor Ferrostatin‐1 and Nrf2 activator Oltipraz could significantly ameliorate the progress of PIO. Here, we identified a new mechanism by which the wear particles downregulated the expression of the Nrf2‐ARE signalling pathway and induced ferroptosis in osteoblasts, which may trigger a decrease in bone formation and promote subsequent peri‐implant osteolysis. Our study provides more insights into the cell death pathways in wear‐particle‐induced osteolysis and suggests a novel therapeutic approach for patients with PIO.

## 2 MATERIALS AND METHODS

### 2.1 Reagents

Penicillin, trypsin‐EDTA, streptomycin, and dimethyl sulfoxide (DMSO) were obtained from Sigma‐Aldrich (St. Louis, MO, USA). RIPA lysis buffer and bovine serum albumin (BSA) were purchased from Beyotime (Shanghai, China). Protease inhibitor cocktail, phosphatase inhibitor cocktail, 4’,6‐diamidino‐2‐phenylindole (DAPI) dye, and trypsin‐EDTA were acquired from Thermo Fisher Scientific (Scoresby, Vic, Australia). Minimum essential medium α (α‐MEM) and foetal bovine serum (FBS) were acquired from Gibco (Grand Island, NY, USA). Ferrostatin‐1 (MF: C_15_H_22_N_2_O_2_, MW: 262.35), RSL3 (MF: C_23_H_21_CIN_2_O_5_, MF: 440.88) and Oltipraz (MF: C_8_H_6_N_2_S_3_, MW: 226.34) were purchased from MedChem Express (Shanghai, China).

### 2.2 Nanoparticle preparation and characterization

CoCrMo nanoparticles (CoNPs), which had an average particle diameter of 90.87 nm, were ground and supplied by Nanjing Kester Metal Products Company. CoNPs were stocked at a concentration of 50 mg/ml in phosphate‐buffered saline (PBS). They were resuspended well in PBS after sonication for 15 min. The CoNPs were autoclaved prior to treatment. The stock solution of CoNPs was diluted to a specific concentration and ultrasonicated for 15 min prior to being exposed to the cells. A field emission scanning electron microscope (Merlin, Carl Zeiss AG, Germany) and transmission electron microscope (JEM‐2000, Japan) were used to determine the morphology of the CoNPs. The size distribution was determined by dynamic light scattering (DLS) using a BI‐200SM multiangle dynamic/static laser scattering instrument (Brookhaven, USA). The chemical composition of the CoNPs was performed using X‐ray photoelectron spectroscopy (XPS) with a photoelectron spectroscopy system (PHI 5000 Versa Probe II, ULVAC‐PHI).

### 2.3 Cell culture

MC3T3‐E1 cells were obtained from the Cell Bank of Chinese Academy of Sciences (Shanghai, China). MC3T3‐E1 cells were maintained in α‐MEM containing 10% FBS, 100 μg/ml streptomycin, and 100 U/ml penicillin in a cell incubator with 5% CO_2_ at 37°C.

### 2.4 RNA‐seq

The MC3T3‐E1 cells were stimulated by CoNPs (50 μg/ml) for 24 h, and total RNA was obtained using TRIzol Reagent. The extracts were screened and amplified using PCR and sequenced using the Illumina HiSeq 4000 instrument. For bioinformatics analysis, raw reads which contained the adapter or had low quality (*Q*‐value ≤20) were deleted and then located in the mouse‐related genome using HISAT2 (version 2.1.0). Samtools were used to sequence the resulting files, and HTSeq (version 0.9.0) analysis was performed to determine the count of every gene.^35^ Here, we used FDR <0.05 and log 2 (fold change) >2 as thresholds to define substantial differentially expressed genes (DEGs). We performed Gene Ontology (GO) and Kyoto Encyclopaedia of Genes and Genomes (KEGG) pathway analyses for further study. Gene set enrichment analysis (GSEA) and bioinformatics analysis were carried out by OmicStudio tools (https://www.omicstudio.cn/tool).

### 2.5 Periprosthetic osteolytic bone tissues

Periprosthetic osteolytic bone tissue was obtained from patients who underwent aseptic loosening after THA. Cases of local infection were carefully ruled out. The clinical data and preoperative X‐ray examinations of the patients are shown in Table 1 and Figure 3A. Samples from patients with developmental dysplasia of the hip (DDH) who underwent THA were used as controls. Unloose specimen was collected from a patient who underwent THA 5 years back and needed further surgery for mechanical loosening due to a tumbling accident. Samples for cases 1–4 were collected from patients who underwent joint revision surgery for aseptic loosening. All the patients had undergone THA at the Department of Orthopedics of Shanghai General Hospital of Shanghai JiaoTong University School of Medicine (Shanghai, China) between 2019 and 2020. The study was approved by the Institutional Research Ethics Committee of Shanghai General Hospital (2021KY076). Every patient signed a written informed consent from the Institutional Research Ethics Committee of Shanghai General Hospital.

**TABLE 1:** Clinical data of patients

Case	Gender	Age (years)	Preoperative diagnosis	Years after implantation	Type of fixation	Specimen collection site
Control	F	55	Hip dysplasia	–	–	Acetabulum
Unloose	F	63	Mechanical loosening	5	Cementless	Acetabular cup
Case1	F	67	Aseptic loosening	17	Cementless	Acetabular cup
Case2	F	47	Aseptic loosening	14	Cementless	Acetabular cup
Case3	F	70	Aseptic loosening	10	Cementless	Acetabular cup
Case4	F	72	Aseptic loosening	12	Cementless	Acetabular cup

Note

F, female, M, male, Unloose, unloose specimen, Case1–4, loose specimens 1–4. Unloose and Case1–4 specimens were collected from the peri‐implant bone tissue and the artificial femoral head.

### 2.6 Total RNA isolation and real‐time PCR

TRIzol reagent was applied for collecting total RNA from MC3T3‐E1 cells, according to the manufacturer's instructions, for different treatments at the indicated time points. PrimeScript RT Master Mix (TaKaRa, Beijing, China) was used to reverse transcribe RNA to cDNA. The mRNA expression levels of the target genes were standardized with GAPDH. Primers for the related genes utilized for real‐time PCR are shown in Table S1.

### 2.7 Cell viability assay

The Cell Counting Kit‐8 (CCK‐8) assay (Dojindo, Japan) was used to test MC3T3‐E1 cell survivability. MC3T3‐E1 cells were cultured in 96‐well plates at a density of 6 × 10^3^ cells per well. Briefly, after different treatments (0, 25, 50, 75, and 100 μg/ml) of CoNPs for 24 h, 10 μl of CCK‐8 were put into a 96‐well plate and incubated at 37°C for 2 h. The optical density (OD) at 450 nm was measured by a microplate photometer.

### 2.8 Western blotting

We used western blotting to analyze the protein expression. Proteins were extracted from MC3T3‐E1 cells through ice‐cold radioimmunoprecipitation assay lysis buffer after different treatments. The BCA protein assay (Beyotime) was applied to determine protein concentrations. The proteins were transferred onto polyvinylidene difluoride membranes. The membranes were blocked with TBS containing 5% skimmed milk, incubated in 0.1% Tween‐20 (TBS‐T) for 1 h, and incubated with a primary antibody at 4°C overnight. After being incubated with a horseradish peroxidase‐conjugated secondary antibody at room temperature for 1 h, membranes were visualized via an enhanced chemiluminescence (ECL) kit. The following primary antibodies were put into use: SLC7A11 (26864–1‐AP, Proteintech), Nrf2 (16396–1‐AP, Proteintech), HO‐1 (10701–1‐AP, Proteintech), NQO‐1 (11451–1‐AP, Proteintech), GPX4 (ab125066, Abcam), COX2 (ab62331, Abcam), ACSL4 (ab155282, Abcam), Keap1 (ab227828, Abcam), α‐tubulin (3873, Cell Signalling Technology), GAPDH (60004–1‐lg, Proteintech), β‐actin (66009–1‐lg, Proteintech), and histone H3 (4499, Cell Signalling Technology). ImageJ software was adopted to quantify western blot.

### 2.9 Nuclear and cytoplasmic extraction

MC3T3‐E1 cells were treated with varying concentrations (0, 25, 50, 75, and 100 μg/ml) of CoNPs for 24 h. To extract cytoplasmic along with nuclear proteins, a nuclear and cytoplasmic extraction kit (Beyotime) was adopted to process cell pellets in line with the instructions of the manufacturer.

### 2.10 ROS analysis

To measure the lipid ROS produced by MC3T3‐E1 cells, we used the live‐cell analysis reagent, BODIPY 581/591 C11 (D3861, Invitrogen, USA). After processing, the transplanted MC3T3‐E1 cells were incubated with the kit reagent at a working concentration of 5 μM for 30 min at 37°C in an incubator. Furthermore, the level of ROS in MC3T3‐E1 cells was determined using 10 μM dichloro‐dihydro‐fluorescein diacetate (DCFH‐DA, Beyotime) in the dark for 20 min at 37°C. To analyze the ROS levels, we used a FACSCalibur flow cytometer equipped with CellQuest Pro (BD Biosciences, Franklin Lakes, USA).

### 2.11 Lipid peroxidation assay and GSH assays

To assess the end product of lipid peroxidation and malondialdehyde (MDA), we used the lipid peroxidation (MDA) assay kit (Jiancheng, Nanjing, China). The cells were homogenized in MDA lysis buffer on ice and centrifuged at 13,000 × *g* for 3 min. The MDA‐TBA adduct was generated when the MDA in the cell lysate reacted with thiobarbituric acid (TBA) solution, of which the chromaticity (OD = 532 nm) was proportional to the amount of MDA present. We measured the relative GSH concentration in cell lysates employing a glutathione assay kit (Sigma, USA) in accordance with the manufacturer's instructions.

### 2.12 Immunofluorescence staining

After treatment under different conditions, the MC3T3‐E1 cells were fixed with 4% paraformaldehyde (PFA) at room temperature for 20–30 min. The cells were then permeabilized with 0.5% Triton X‐100 for 15 min. After blocking in 1% bovine serum albumin (BSA) for 30 min, the cells were incubated overnight with primary antibody at 4°C. The dilution ratio of Nrf2 for immunofluorescence staining is 1:200 and the dilution ratio of GPX4 is 1:100. The cells were then washed thrice for 10 min in phosphate‐buffered saline (PBS) and incubated with red‐fluorescent Alexa Fluor 594 rabbit anti‐mouse IgG (Invitrogen) for visualization. After being washed in PBS and stained with DAPI for 3 min, the cells were photographed under a fluorescence microscope (Olympus FluoView^TM^ FV1000, Tokyo, Japan).

### 2.13 Transient transfection

Double‐stranded small interfering RNA against Keap1 was used in this study. RiboBio Co., Ltd (Guangzhou, China) designed and chemically synthesized Keap1 siRNAs (si‐keap1). The sequences of si‐keap1 were as follows: si‐keap1‐1: 5’‐CCAATTCATGGCTCACAAA‐3’; si‐keap1‐2: 5’‐GCATCGACTGGGTCAAATA‐3’; si‐keap1‐3: 5’‐CGAATGACATCGGGCCGGA‐3’. Briefly, we cultured the cells in 6‐well plates prior to transfection. At about 85% confluence, the cells were transfected with Lipofectamine 3000 (Invitrogen) in accordance with the manufacturer's instructions. The cells were cultivated for 24 h after transfection before use in subsequent tests.

### 2.14 Particle ‐induced osteolysis animal model

A surgically induced calvaria resorption model using CoNPs was used to study the pathogenesis of osteolysis. In summary, 25 10‐week‐old male C57BL/6 mice were obtained from Slac Laboratory Animal Co. Ltd. and housed in a specific pathogen‐free facility at the Laboratory Animal Department of Shanghai General Hospital. The mice were divided into five groups (*n* = 5), and different groups received treatments as follows: Group I: treated with PBS as controls; Group II: CoNPs; Group III: CoNPs plus Ferrostatin‐1 co‐stimulation; Group IV: CoNPs plus Oltipraz co‐stimulation; Group V: CoNPs plus Ferrostatin‐1 and Oltipraz co‐stimulation. Specifically, the male C57BL/6J mice were anesthetized by the intraperitoneal injection of 4% chloral hydrate (0.25 ml per kg body weight). After anesthetization, the skulls of the mice were exposed through midline sagittal incisions of 15 mm on the head. Forty microliters of metal particle suspensions (50 mg/ml) in PBS were embedded into the surface of the bilateral parietal bones. In group I, the middle of the calvariae was carpeted with 40 μl of PBS as a control. In Group II, the mice took 40 μl of 50 mg/ml CoNPs. In Group III, the mice received 40 μl of 50 mg/ml CoNPs and were pre‐treated with 1 mg/kg Ferr‐1 by intraperitoneal injection. In Group IV, the mice received 40 μl of 50 mg/ml CoNPs and were pre‐treated with 0.8 mg/kg Oltipraz by intraperitoneal injection. In Group V, the mice received 40 μl of 50 mg/ml CoNPs and were pre‐treated with 1 mg/kg Ferr‐1 and 0.8 mg/kg Oltipraz by intraperitoneal injection. Ferrostatin‐1 or Oltipraz was injected every two days for two weeks before sacrifice. There were no deaths or complications up to 7 days after the operation. All steps were implemented in accordance with animal welfare procedures and with permission from the Research Ethics Committee of Shanghai General Hospital (#2020AW117).

### 2.15 Micro‐CT analysis

After harvesting, the mouse calvaria were analyzed with high‐resolution micro‐CT (YUEBO Company, Hangzhou, China) at a resolution of 10 μm (70 kV, 200 μA). 3D images of a square region of interest besides the midline suture were reconstructed. Structure model index (SMI), trabecular thickness (Tb. Th), trabecular number (Tb.N), bone mineral density (BMD), bone volume/total tissue volume (BV/TV), and trabecular separation (Tb. Sp) were measured using the manufacturer's software.

### 2.16 Histological assessments

All C57BL/6 mice were euthanized 2 weeks after surgery. The mouse calvaria were fixed in 4% PFA for 24 h, decalcified with 10% EDTA for one month, and embedded in paraffin. The calvaria was sliced into 4‐μm‐thick sections and stained with haematoxylin and eosin (H&E).

### 2.17 Statistical analysis

All data represent means ± SD of triplicate independent experiments. Data analysis was performed by SPSS software (version 22.0; SPSS, Chicago, USA). T‐tests or a one‐way ANOVA were used to determine the differences between various groups. (^*,#^ indicate *p* < 0.05, ^**,##^ indicate *p* < 0.01, ^ns^indicates not significant).

## 3 RESULTS

### 3.1 Physical characteristics of CoNPs

**FIGURE 1:**
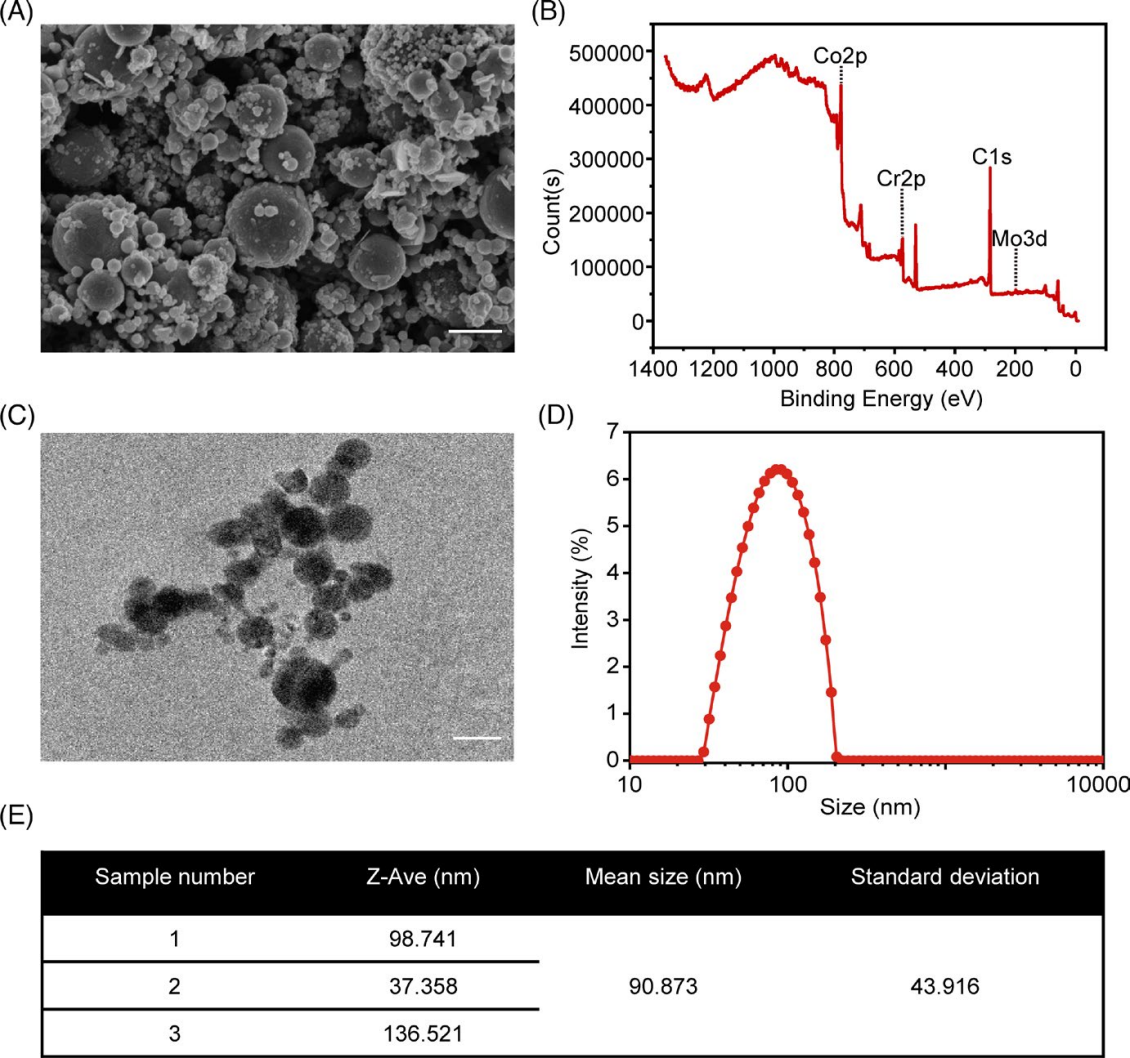
Physical characteristics of CoCrMo nanoparticles. (A) Scanning electron microscopy (SEM) image of CoNPs. Scale bar: 100 nm. (B) Chemical composition of CoNPs, using X‐ray photoelectron spectroscopy (XPS). (C) Transmission electron microscopy (TEM) image of CoNPs. Scale bar: 100 nm. (D) The size distribution of CoNPs, and (E) their mean size

Scanning electron microscopy (SEM) image (Figure 1A) and transmission electron microscopy (TEM) image (Figure 1C) performed the appearance of CoNPs, which varied in size and were basically spherical. Figure 1D shows further size distribution analysis using a Nano Sizer. The size of particles ranged between 29.30 nm and 204.27 nm, and the mean particle diameter was 90.87 nm (Figure 1E). The chemical composition of the CoNPs was performed using XPS, and the main composition of CoNPs was Co2p, Cr2p and Mo2d, as shown in Figure 1B.

### 3.2 RNA‐seq analysis revealed that CoNPs downregulated GPX4 expression in MC3T3‐E1, which correlates with ferroptosis

**FIGURE 2:**
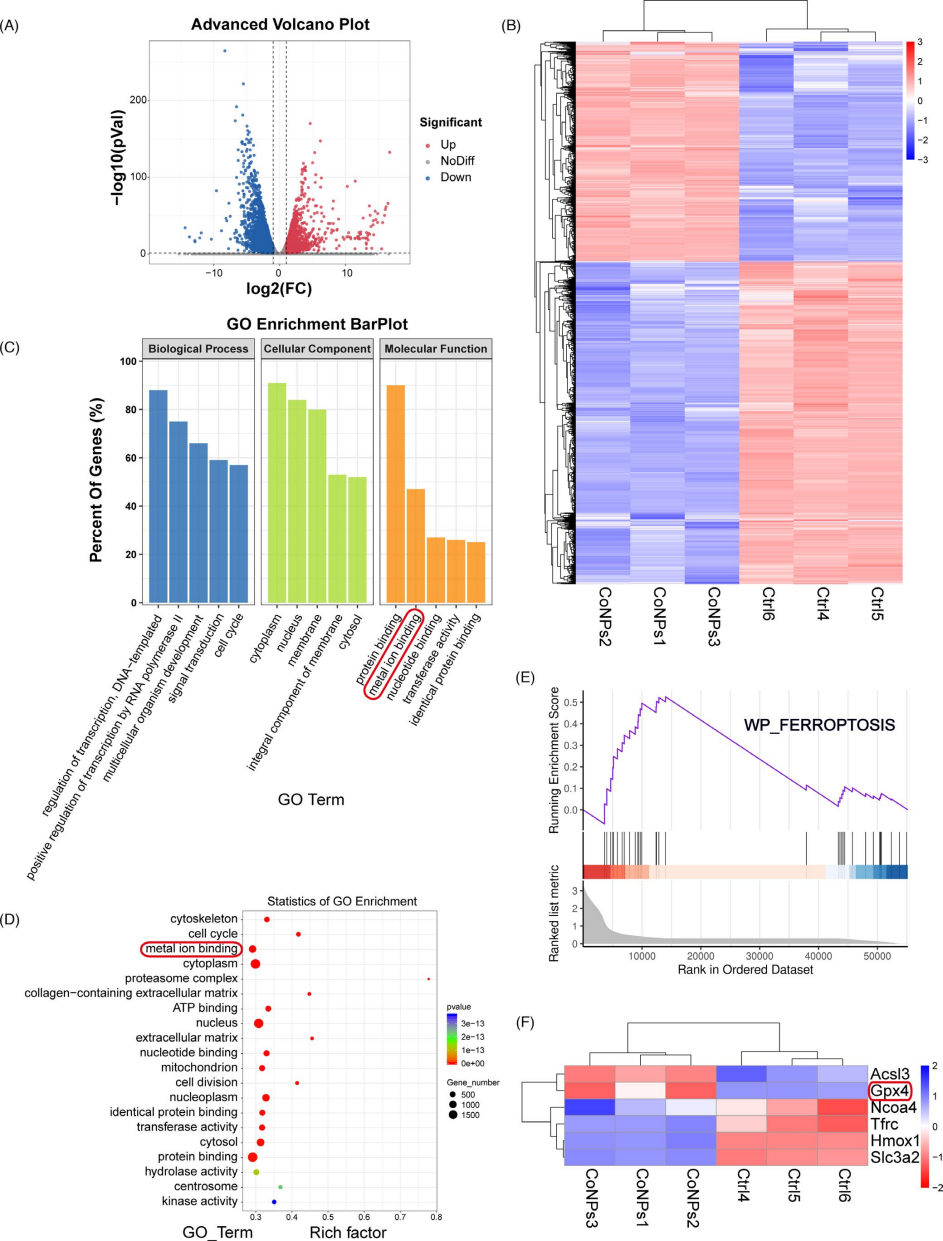
RNA‐seq analysis revealed that CoNPs downregulated GPX4 expression in MC3T3‐E1 cells, which correlates with ferroptosis. (A) Volcano plot reveals 2358 upregulated genes and 3458 downregulated genes in MC3T3‐E1 cells after CoNPs (50 μg/ml) stimulations for 12 h. (B) Heat map and cluster analysis. (C, D) Significant pathway of GO Enrichment analysis in which metal ion binding enriched significantly. (E) Enrichment plot of Ferroptosis gene sets which were importantly differentiated between the control group and CoNPs group. (F) Heat map and cluster analysis between Ferroptosis gene sets and differential genes in which ferroptosis core regulator GPX4 decreased significantly

We isolated the total mRNA of MC3T3‐E1 osteoblasts after 50 μg/ml CoNPs treatment for 12 h, and the RNA‐seq was analyzed using the Illumina HiSeq 2500 platform to generate mRNA profiles. The volcano plot revealed 2358 upregulated genes and 3458 downregulated genes in MC3T3‐E1 after CoNPs (50 μg/ml) stimulations for 12 h (Figure 2A). A heat map was plotted, and cluster analyses were performed (Figure 2B). The 5816 differentially expressed genes (DEGs) were annotated using GO categories (Figure 2C and 2D) and KEGG pathway analysis (Figure S1). Significant pathways from the GO Enrichment analysis showed that metal ion binding enriched significantly in the molecular function group, which may correlate with ferroptosis.^36^ Then, ferroptosis gene sets were downloaded from the Molecular Signatures Database (MSigDB). GSEA was performed to analyze ferroptosis gene sets, which differed significantly between the control group and CoNPs‐treated group (Figure 2E). Heat map and cluster analysis between ferroptosis gene sets and DEGs have shown that the level of ferroptosis core regulator, GPX4, decreased significantly, which marked the occurrence of ferroptosis(Figure 2F).^37^ In brief, our RNA‐seq analysis results suggest that ferroptosis occurs in MC3T3‐E1 cells after treatment with CoNPs.

### 3.3 Activation of ferroptosis and suppression of osteoblast in peri‐implant bone tissue of patients with aseptic loosening

**FIGURE 3:**
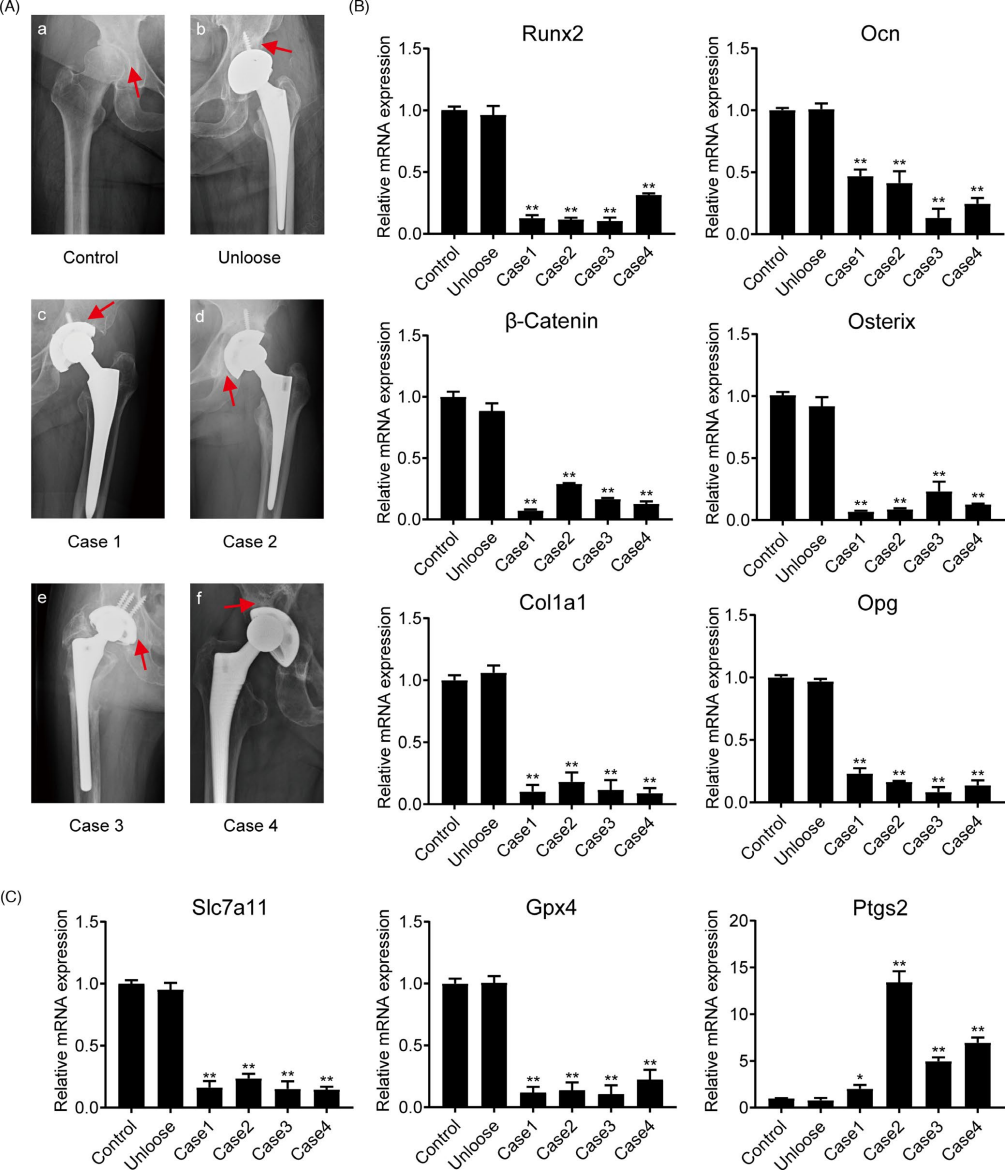
Preoperative examination of x‐rays from patients with aseptic loosening and the analysis of related mRNA expression in peri‐implant bone tissue of patients. (A) Preoperative radiographs of patients with aseptic loosening. The red arrow represents the site from where we collected the specimens. Control: developmental dysplasia of the hip required THA. Unloose: unloose specimens; Case1‐4: loose specimens. (B) The mRNA expression of osteogenic marker genes Runx2, Ocn, β‐Catenin, Osterix, Col1a1, and Opg in bone tissue of these patients. (C) The mRNA expression of ferroptosis‐related genes, Gpx4, Slc7a11, and Ptgs2, in bone tissue from these patients. Data represent mean ± SD of triplicate independent experiments. ^*, #^ indicates *p* < 0.05, ^**, ##^ indicates *p* < 0.01, ^ns^ indicates not significant

Preoperative radiographs of the patients are shown in Figure 3A. Previous studies have shown that the degree of PIO assessed by intraoperative discovery was consistent with preoperative X‐rays and the implantation time of the revision patient.^38^, ^39^ From preoperative X‐rays, we can see that there is a black signal between the implant and peri‐implant bone tissue, which means the bone tissue around the implant has been damaged and the prosthesis is loosening. Previous studies have revealed that CoNPs‐induced PIO by promoting osteoblast apoptosis.^11^, ^40^ We used real‐time PCR assay to determine the content of osteoblast markers in peri‐implant bone tissues. Compared with the control, the expression of Runx2, Ocn, β‐Catenin, Osterix, Col1a1, and Opg decreased as shown in Figure 3B. Furthermore, RNA‐seq transcriptome analysis revealed that CoNPs are capable of inducing ferroptosis in MC3T3‐E1 osteoblasts. To investigate whether ferroptosis is involved in the process of osteolysis in patients with AL, an mRNA assay of ferroptosis‐related genes in clinical unloose specimens and peri‐implant bone tissue was performed. The mRNA expression of Gpx4 and Slc7a11 significantly downregulated in Case1–4, and the expression of Ptgs2 increased in Case1–4 relative to the control (Figure 3C). Taken together, these data confirmed the activation of ferroptosis and suppression of osteoblasts in peri‐implant bone tissue of patients with AL.

### 3.4 CoNPs‐induced osteoblasts ferroptosis in vitro

**FIGURE 4:**
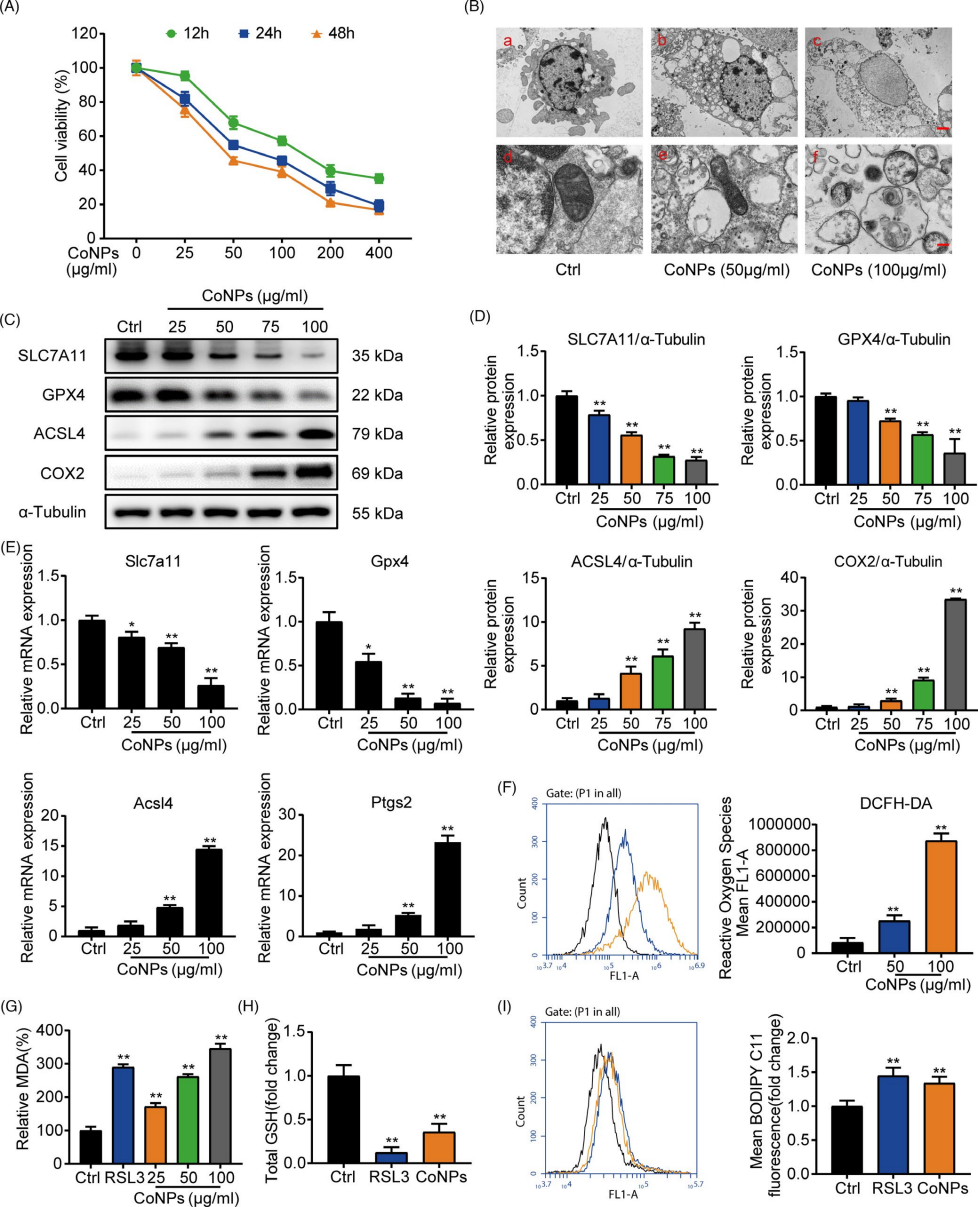
CoNPs‐induced osteoblasts ferroptosis in vitro. (A) Effect of gradient concentrations (0, 25, 50, 100, 200, 400 μg/ml) of CoNPs on the osteoblast survival was observed by CCK‐8. (B) Transmission electron microscopy of osteoblasts after treatment with gradient concentrations of CoNPs for 24 h. Scale bar: 20 μm (upper) and 2 μm (lower). (C) Western blots performed after MC3T3‐E1 cells were treated with CoNPs for 24 h (*n* = 3). (D) ImageJ software was used to quantify the density of the western blot bands shown in (C). (E) Relative mRNA expression of Gpx4, Slc7a11, Acsl4, and Ptgs2 in each group. (F) ROS generation was detected by flow cytometry with DCFH‐DA (10 mM) in each group. (G) Lipid peroxide MDA level in each group. (H) The GSH level in each group. (I) Cell lipid peroxidation was detected by BODIPY 581/591 C11 staining using flow cytometry in each group. ^*, #^ indicates *p* < 0.05, ^**, ##^ indicates *p* < 0.01, ^ns^ indicates not significant

For sake of exploring the role of CoNPs in osteoblasts, the effect of CoNPs stimulation on the survivability of osteoblasts was verified. As shown in Figure 4A, with the increase of CoNPs stimulation time or concentrations, the number of osteoblasts decreased gradually. In subsequent studies, we selected the appropriate CoNPs concentration and stimulation time to treat osteoblasts. Transmission electron microscopy images of cell mitochondria showed the occurrence of ferroptosis. As presented in Figure 4B, compared with controlled‐treated osteoblasts, cells treated with CoNPs (50 μg/ml) showed smaller, crumpled, and fractured mitochondria, and the cristae of mitochondria disappeared.^41^ Cells treated with CoNPs (100 μg/ml) changed more prominently. Several key factors, such as SLC7A11, GPX4, ACSL4, and COX2, are considered as key proteins involved in ferroptosis regulation.^12^ Hence, to assess ferroptosis sensitivity after MC3T3‐E1, the cells were treated with CoNPs in vitro. We verified the content of the proteins under different concentrations of CoNPs (0, 25, 50, 75 and 100 μg/ml). Figure 4C and D displayed that the protein content of ACSL4 and COX2 increased in the cells, while the content of GPX4 and SLC7A11 decreased after 24 h of treatment with CoNPs. Meanwhile, a similar trend of these core factors was seen in the osteoblast cells treated with CoNPs (50 μg/ml) in a time gradient (0, 6, 12, 24 and 48 h), as shown in Figure S2A and B. In addition, according to Figure 4E and Figure S2C, real‐time PCR analysis of Slc7a11, Gpx4, Acsl4, and Ptgs2 presented a similar trend as that observed in Figure 4C and Figure S1. Furthermore, lipid peroxidation, which can result in ROS accumulation, is one of the hallmarks of ferroptosis.^42^ We used fluorescent probes, DCFH‐DA and C11‐BODIPY, to detect the content of ROS and lipid peroxidation in osteoblasts by flow cytometry.^43^ As shown in Figure 4F and I, compared with control‐treated osteoblasts, osteoblasts exhibited an upregulation in fluorescence after 24 h stimulation with CoNPs (50 and 100 μg/ml), which had a similar effect as the ferroptosis activator, RSL3.^44^ Furthermore, the content of malondialdehyde (MDA) was detected, which is the end product of lipid oxidation.^36^ As shown in Figure 4G, CoNPs caused an obvious upregulation in MDA levels in osteoblasts, similar to RSL3.^44^ Finally, the GSH content in osteoblasts was quantified after treatment of CoNPs (Figure 4H). There was a clear downregulation in CoNPs‐treated osteoblasts compared with that in control‐treated cells. In conclusion, the data showed that CoNPs triggered ferroptosis in osteoblast cells in vitro.

### 3.5 Ferrostatin‐1 ameliorates the ferroptosis induced by CoNPs in vitro

**FIGURE 5:**
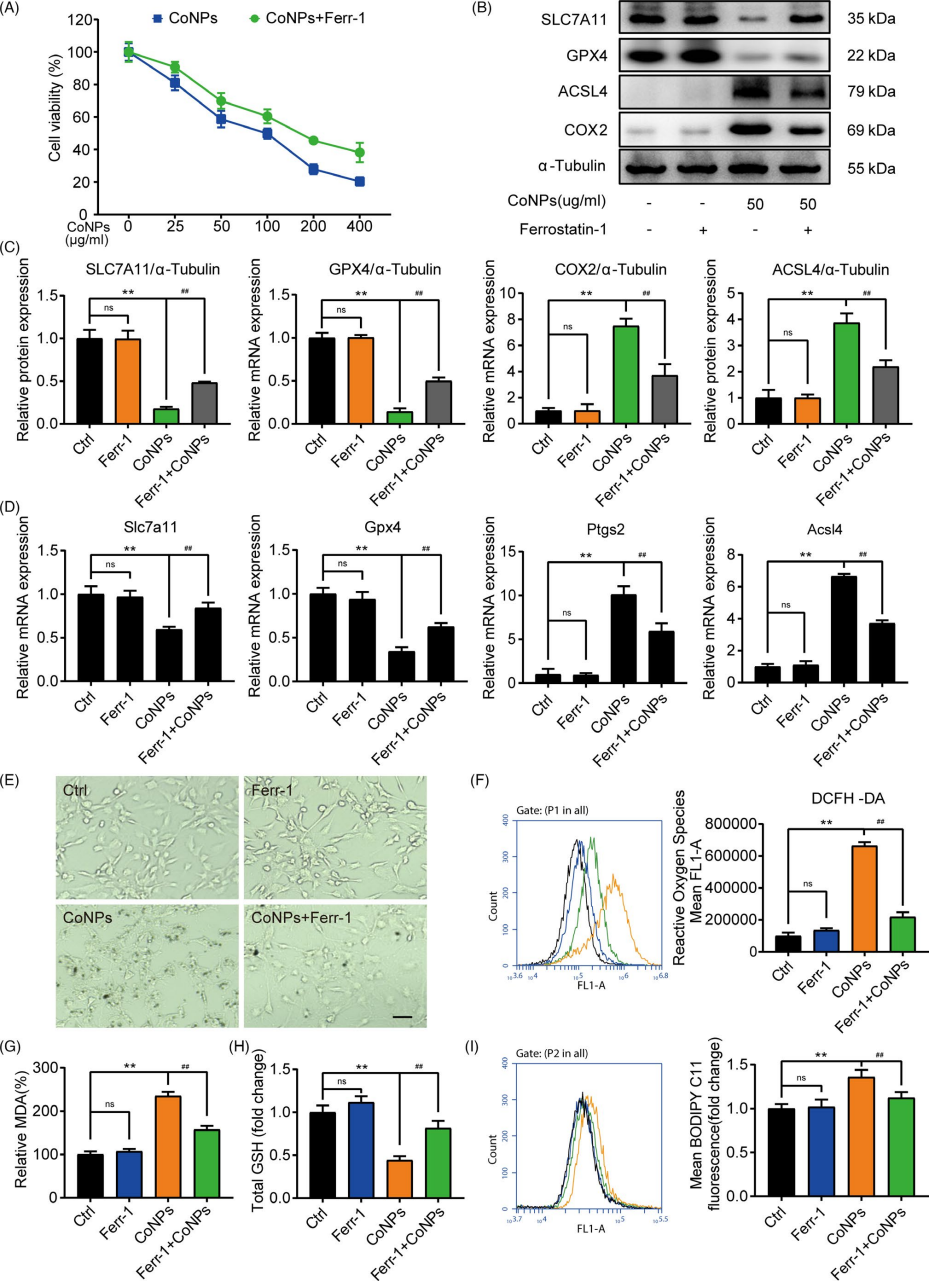
Ferrostatin‐1 protected osteoblasts against ferroptosis induced by CoNPs in vitro. (A) Osteoblasts were treated with ferrostatin‐1 (1 μM/ml) for 12 h before treatment with CoNPs for 24 h. Cell viability was observed by CCK‐8. (B) Western blots performed after osteoblasts being stimulated with indicated treatment. (C) The quantification of the western blot bands shown in (B). (D) Relative mRNA expression in each group. (E) Light microscopic changes of MC3T3‐E1 cells in each group. Scale bar: 100 μm. (F) ROS generation was demonstrated by flow cytometry with DCFH‐DA (10 mM) in each group. (G) Lipid peroxide MDA level in each group. (H) The GSH level in each group. (I) Cell lipid peroxidation was detected by BODIPY 581/591 C11 staining in each group. ^*, #^ indicates *p* < 0.05, ^**, ##^ indicates *p* < 0.01, ^ns^ indicates not significant

Ferrostatin‐1 (Ferr‐1), the inhibitor of ferroptosis, which could rescue the injury specifically, was used to determine the appearance of ferroptosis in osteoblasts after treatment of CoNPs.^45^ Previous studies have shown that Ferr‐1 can suppress ferroptosis in different cells.^46^, ^47^ Ferr‐1 can prevent ferroptosis by directly inhibiting the production of reactive oxygen species.^12^ CCK8 kit was used to confirm the concentration of Ferr‐1 that suppressed osteoblast proliferation and viability (Figure S3A). Compared with the control group, up to 4 μM/ml Ferr‐1 had no obvious cytotoxic effect on osteoblasts. Another CCK8 assay was performed to verify whether Ferr‐1 could ameliorate the ferroptosis in osteoblasts induced by CoNPs. As shown in Figure 5A, pre‐treating osteoblasts with Ferr‐1 (1 μM/ml) for 12 h before treatment with CoNPs distinctly improved the cell viability compared with that of the cells treated directly with CoNPs. The results from the western blot assay show that CoNPs treatment significantly downregulated the content of SLC7A11and GPX4 and upregulated the content of ACSL4 and COX2, while Ferr‐1 inhibited the degradation of SLC7A11 and GPX4 and upregulated the expression of ACSL4 and COX2 in Figure 5B and C. Moreover, as presented in Figure 5D, we performed real‐time PCR analysis of Slc7a11, Gpx4, Acsl4, and Ptgs2. The analysis showed the same results as Figure 5B and C. Furthermore, light microscopy showed that compared with control‐treated osteoblasts, cells treated with CoNPs (50 μg/ml) shrank and their cell membranes ruptured. However, Ferr‐1 rescued cell morphology and suppressed the ferroptosis induced by CoNPs (Figure 5E). Next, we analyzed cellular ROS and lipid peroxidation in osteoblasts using DCFH‐DA and C11‐ BODIPY, respectively. As shown in Figure 5F and I, the change in fluorescence showed that CoNPs treatment obviously increased the cellular ROS and lipid peroxidation, whereas Ferr‐1 decreased the generation of ROS and lipid peroxidation in osteoblasts pre‐treated with Ferr‐1 for 12 h. We further quantified the end product of lipid oxidation, malondialdehyde (MDA), and GSH content. As shown in Figure 5G and H, Ferr‐1 reduced the production of MDA and suppressed the degradation of GSH. Collectively, Ferrostatin‐1 suppressed the ferroptosis induced by CoNPs in vitro.

### 3.6 The expression of Nrf2 decreased in the osteoblasts after treatment with CoNPs

**FIGURE 6:**
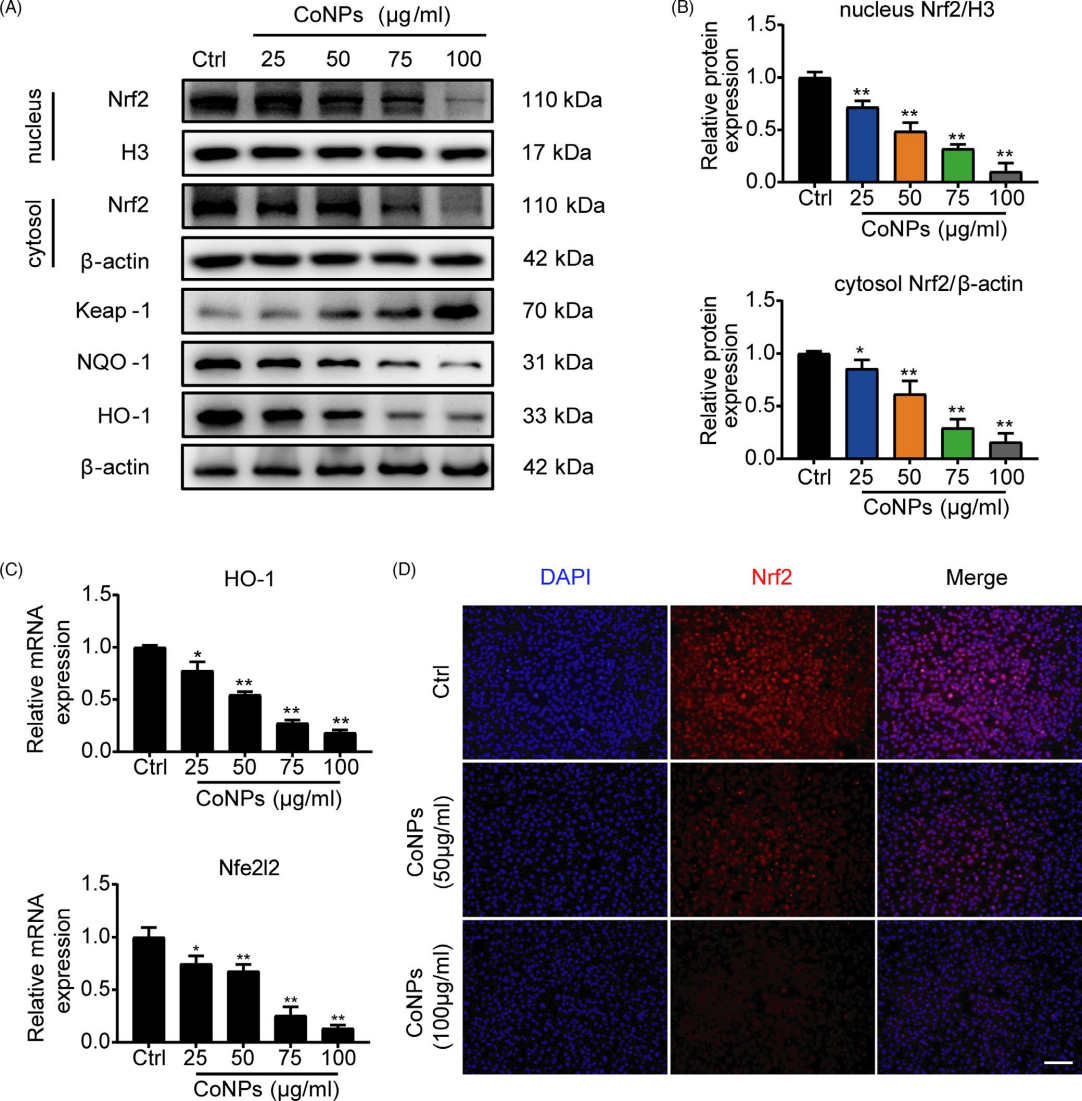
CoNPs downregulated the expression of Nrf2 in vitro. (A) Western blots performed after osteoblasts were treated with gradient concentrations (0, 25, 50, 75, 100 μg/ml) of CoNPs for 24 h (*n* = 3). (B) The quantification of the western blot bands shown in (A). (C) Relative mRNA expression of Nfe2l2 and HO‐1 in each group. (D) Immunofluorescence staining of Nrf2 in MC3T3‐E1 cells treated with CoNPs (50 μg/ml). Scale bar: 100 μm. Data represent means ± SD of triplicate independent experiments. ^*, #^ represent *p* < 0.05, ^**, ##^ represent *p* < 0.01

Nrf2, the nuclear factor erythroid 2‐related factor 2, has been confirmed to maintain cell homeostasis against oxidative stress induced by various types of cell death,^29^ such as ferroptosis, apoptosis, and autophagy, but whether CoNPs‐induced ferroptosis in osteoblasts by downregulating Nrf2 remains to be studied. To determine the expression of Nrf2 in the osteoblasts, results from the western blot assay showed that as the stimulation concentration of CoNPs increased, the content of Nrf2 in the cell nucleus and the cytosol significantly decreased (Figure 6A and B). Moreover, the mRNA level of Nrf2 decreased (Figure 6C). Immunofluorescence staining of Nrf2 in osteoblasts was performed after treatment with gradient concentrations of CoNPs. Figure 6D shows a decrease in Nrf2 expression in osteoblasts after treatment of CoNPs compared with those in the control group. Previous research has shown that Nrf2 regulates cellular redox homeostasis by binding its promoter with target genes that have antioxidant response elements (AREs),^17^, ^31^ such as heme oxygenase‐1 (HO‐1) and quinone oxidoreductase‐1 (NQO‐1). Thus, we analyzed the expression of AREs, and the results of the western blot assay showed a decrease in HO‐1 and NQO‐1 expression after treatment with CoNPs (Figure 6A). The mRNA expressions of HO‐1 decreased in a similar manner (Figure 6C). The data indicate that the expression of Nrf2 was lowered in the osteoblasts after being treated with CoNPs.

### 3.7 CoNPs‐induced osteoblasts ferroptosis via regulating Nrf2‐ARE signalling pathway

**FIGURE 7:**
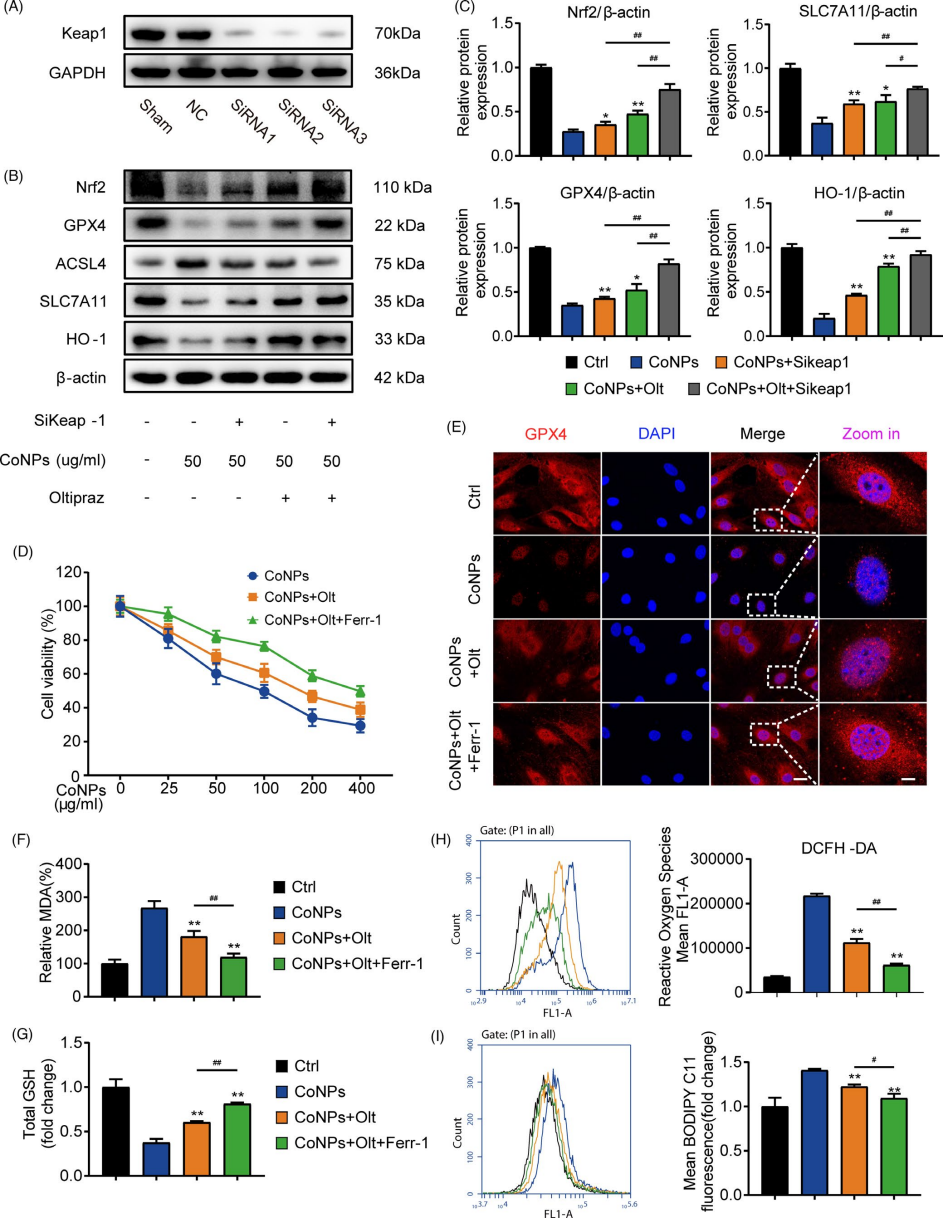
Oltipraz suppresses ferroptosis via activation of the Nrf2‐ARE signalling pathway. (A) Western blots performed after cells were incubated with sham, siControl, and siKeap1. (B) Western blots performed after MC3T3‐E1 cells were incubated with indicated treatment. (C) The quantification of the western blot bands shown in (B). (D) Cell survival was determined by CCK‐8. (E) Immunofluorescence of GPX4 expression in MC3T3‐E1 treated with PBS (Control), CoNPs, CoNPs + Olt, and CoNPs + Olt + Ferr‐1. Scale bar: 20 μm (before Zoom in); 2 μm (after Zoom in). (F) The lipid peroxide MDA level in each group. (G) The GSH level in each group. (H) ROS generation was demonstrated by flow cytometry with DCFH‐DA (10 mM) in each group. (I) Cell lipid peroxidation was detected by BODIPY 581/591 C11 staining in each group. ^*, #^ indicates *p* < 0.05, ^**, ##^ indicates *p* < 0.01, ^ns^ indicates not significant

This study shows that CoNPs‐induced osteoblast ferroptosis and decreased the expression of Nrf2, but the link between them remains unclear. Previous study has shown that the proteasomal activity of Kelch‐like ECH‐associated protein 1 (Keap1) decomposed Nrf2 in a durative manner.^29^, ^32^ The suppression of Keap1 activates the Nrf2‐ARE pathway under various oxidative stress conditions.^33^ Sikeap1, an siRNA against Keap1 (Figure 7A), and the Nrf2 specific activator Oltipraz (Olt) were used to activate Nrf2. SiRNA2 of Keap1 was selected for the next study. As shown in Figure S3B, Oltipraz had no significant toxicity on osteoblast viability until cell viability decreased with exposure to up to 20 μM. Treatment with CoNPs (50 μg/ml) significantly upregulated the protein content of ACSL4, and decreased the protein expression of GPX4 and SLC7A11, but pre‐treatment with Sikeap1, Oltipraz, or Sikeap1 plus Oltipraz reversed the tendency (Figure 7B and C). Moreover, confocal microscopy revealed that Oltipraz reversed the decrease in GPX4 expression in osteoblasts stimulated by CoNPs. A combination of Ferr‐1 and Oltipraz could be more effective (Figure 7E). Furthermore, Oltipraz obviously improved the cell viability (Figure 7D), downregulated the percentage of MDA (Figure 7F), and increased the content of GSH (Figure 7G) in osteoblasts treated with CoNPs. The results of fluorescent probes DCFH‐DA and C11‐BODIPY indicated that Oltipraz significantly suppressed the production of cellular ROS and lipid peroxidation (Figure 7H and I), which protected osteoblasts against ferroptosis. The combined utilization of Ferrostatin‐1 + Oltipraz had a more prominent effect than Oltipraz alone. These data indicate that CoNPs induce osteoblast ferroptosis by downregulating the Nrf2‐ARE signalling pathway.

### 3.8 Suppressing ferroptosis and activating Nrf2 signalling pathway ameliorated CoNPs‐induced osteolysis in vivo

**FIGURE 8:**
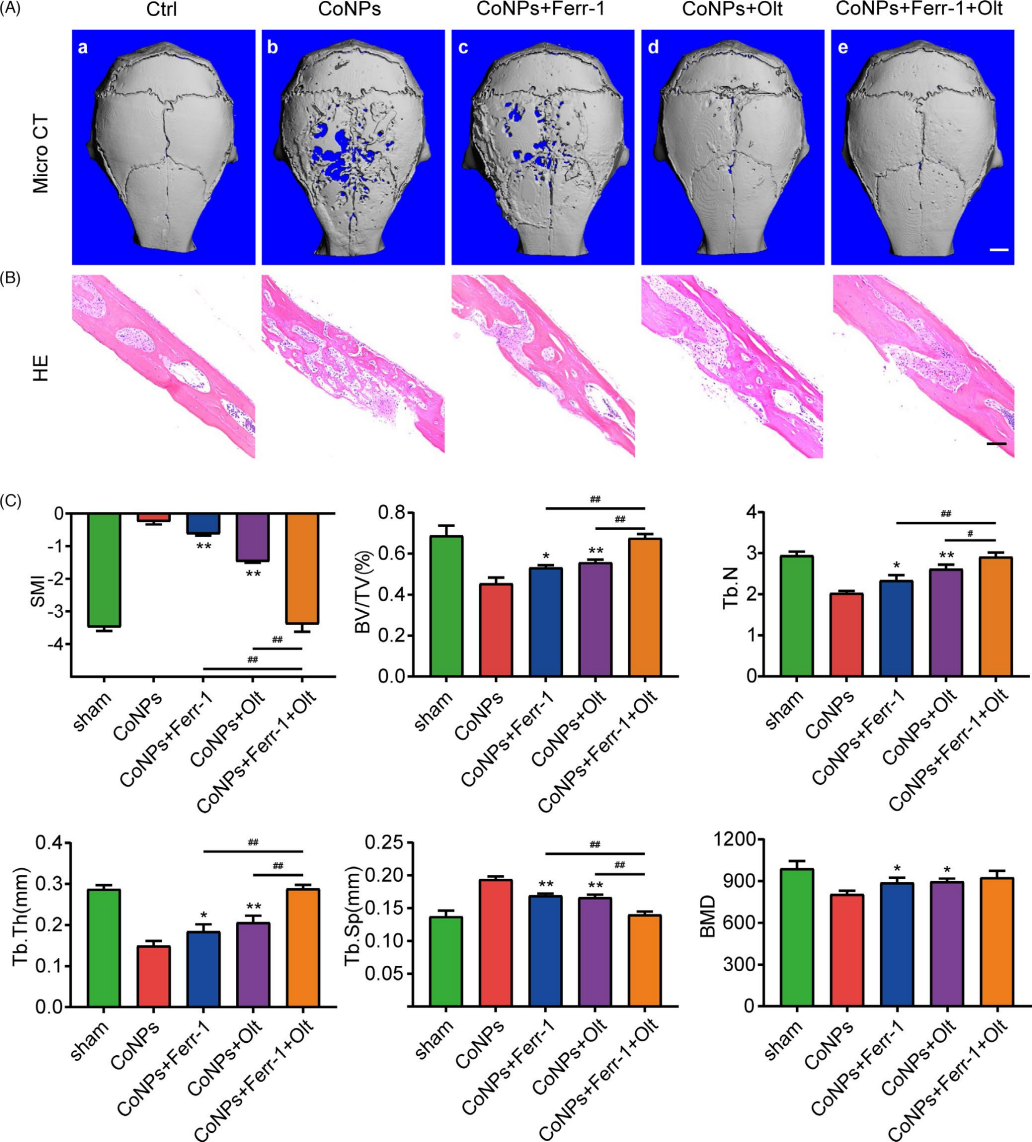
Oltipraz and Ferrostatin‐1 attenuated CoNPs‐induced osteolysis in vivo. (A) 3‐Dimensinal reconstruction micro‐CT images from the outside of the calvaria after treatment. (a–e, representing the sham group, CoNPs group, CoNPs + Ferrostatin‐1 treatment group, CoNPs + Oltipraz treatment, and CoNPs + Ferrostatin‐1 + Oltipraz treatment group, respectively). Scale bar, 2 mm. (B) HE staining of calvaria of the five groups, Scale bar, 50 μm. (C) Quantification of SMI, BV/TV, BMD, Tb. Th, Tb. Sp, Tb.N. Data represent means ± SD of triplicate independent experiments. ^*, #^ represent *p* < 0.05, ^**, ##^ represent *p* < 0.01. ^*, **^ vs. CoNPs; ^#, ##^ vs. CoNPs + Ferr‐1 + Olt; ^ns^ represents not significant

Our analysis confirmed that CoNPs downregulated the Nrf2‐ARE signalling pathway and induced osteoblast ferroptosis in vitro. To further investigate the impact of osteoblast ferroptosis in the pathogenic process of osteolysis, we established a calvaria resorption model with male C57BL/6J mice, which is a frequently used model for the study of AL.^11^ As mentioned above, we divided the mice into five groups. There were five C57BL/6J mice in each group. Micro‐CT with 3‐dimensional reconstruction was used to examine the osteolysis induced by CoNPs. Figure 8A shows that CoNPs‐induced bone loss was suppressed by co‐treatment with ferroptosis inhibitor Ferrostatin‐1 or Nrf2 activator Oltipraz, and combined utilization of Ferrostatin‐1 and Oltipraz dramatically ameliorated the progress of bone loss compared with the control group. SMI, BV/TV, BMD, Tb. Th, Tb.N, and Tb. Sp were measured based on the 3D‐reconstructed images (Figure 8C). Hematoxylin‐eosin (HE) staining analysis showed that treatment with Ferrostatin‐1 or Oltipraz markedly ameliorated the progression of osteolysis induced by CoNPs, and Ferrostatin‐1 plus Oltipraz relieved osteolysis more effectively (Figure 8B). As shown in Figure S3C and D, the treatment of Ferrostatin‐1 or Oltipraz had no significant negative influences on the body weight and major internal organs of mice. These observations suggest that Ferrostatin‐1 and Oltipraz have a strong inhibitory effect on osteolysis in vivo, which may be a new therapeutic approach in PIO.

## 4 DISCUSSION

THA is generally accepted as the most effective surgical method for trauma, degenerative arthritis, and other end‐stage joint diseases. Nevertheless, periprosthetic osteolysis and subsequent AL caused by wear particles frequently lead to the failure of THA and surgical revision, which greatly add to the social burden.^2^ Wear particles, which are generated from numerous implanted prostheses, are the main cause of osteolysis and eventually AL. Previous researches have shown that wear particles can interact with various kinds of cells, including macrophages, osteoblasts, and other mesenchymal cells, at the interface between the implant component and the surrounding bone, which can release a mass of cytokines, chemokines, and metalloproteinases. These mediators influence cell functions, upsetting the balance between bone formation and resorption.^48^ Preceding researches have suggested that the activation of bone resorption is the main cause of osteolysis, which is mainly dependent on osteoclasts and macrophages.^7^ When stimulated by wear particles, inflammatory cytokines, such as TNF‐α, IL‐6, IL‐1β, IL‐6, IL‐8, matrix metallopeptidases (MMPs), and PGE_2_ (prostaglandin E_2_), are released from the cells, leading to bone resorption and indirect osteoclast activation. However, the downregulation of bone formation due to inhibition of osteoblastic function has not attracted sufficient attention. Osteoblasts are in charge of maintaining homeostasis during bone formation and degradation. Osteoblasts secrete type 1 collagen (comprising 90% of the extracellular matrix) and play a key role in matrix mineralization. Wear particles have been proven to affect the function of osteoblasts and promote osteolysis via two distinct mechanisms.^11^ First, proinflammatory mediators are produced by osteoblasts after stimulation by wear particles, such as IL‐6 and TNF, which contributed to a cascade of inflammatory reaction leading to osteolysis around prosthesis. Second, the ability of osteoblasts to secrete mineralized bone matrix, exhibit alkaline phosphatase activity and osteoblast proliferation, was suppressed by wear particles. Although existing studies have demonstrated that osteoblasts are damaged during PIO, this research has been limited to in vitro studies, and the mechanism is not fully clear.^10^ In this study, we found that wear particles caused osteoblast ferroptosis in the process of PIO, which may be a new therapeutic target in AL.

Previous studies have found that there are a variety of programmed cell death mechanisms for primary human osteoblasts and osteoblast‐like cells stimulated by wear particles, including apoptosis and autophagy.^35^ In this study, RNA‐Seq analysis revealed that CoNPs downregulated the expression of GPX4 in osteoblasts, which is of crucial importance in regulating ROS levels. The downregulation of GPX4 tended to promote the accumulation of lipid peroxidation, leading to ferroptosis. Moreover, several studies have proved that ferroptosis can result in the occurrence of multiple types of organic diseases, such as Alzheimer's disease, carcinogenesis, intracerebral haemorrhage, traumatic brain injury, stroke, and ischaemia‐reperfusion injury.^22^ However, the involvement of osteoblast ferroptosis in the progress of PIO remains unclear. Analysis of experimental data from the clinical specimens showed that there was an association between ferroptosis and reduced osteoblasts. Then, our results suggested that osteoblast ferroptosis induced by CoNPs is associated with osteolysis. Transmission electron microscopy revealed that compared with control‐treated osteoblasts, osteoblasts treated with CoNPs (50 μg/ml) showed smaller, crumpled, and fractured mitochondria, and the cristae of mitochondria disappeared. Furthermore, the expression of ferroptosis markers, GPX4, SLC7A11, ACSL4, and COX2 changed significantly. Excessive lipid peroxidation and GSH downregulation indicate the loss of antioxidant capacity. More importantly, we found that the distinct inhibitor Ferrostatin‐1 (ROS‐particular inhibitor) could efficiently reverse particle‐induced ferroptosis both in vivo and in vitro. These consequences confirmed that osteoblast ferroptosis was triggered by CoNPs in PIO.

Considering the appearance of ferroptosis and the reduction in the number of osteoblasts, we focused on elucidating the relationship between ferroptosis and wear particle‐stimulated osteolysis, and investigating the effect of suppressing ferroptosis as a potential approach for the treatment of PIO. The transcription factor Nrf2 has been proved to regulate cellular antioxidant response by expressing many genes that counteract oxidative and electrophilic stresses.^33^ Li et al^49^ proved that Nrf2 activation accelerates impaired wound healing via ameliorating diabetes‐mediated oxidative stress and inflammation. In addition to playing an important role in maintaining cellular redox balance, Nrf2 may also be crucial in preventing ferroptosis directly or indirectly. Dong et al^50^ confirmed that Nrf2 inhibits ferroptosis and prevents acute lung injury on account of intestinal ischaemia reperfusion through regulating SLC7A11 and HO‐1. Jiang et al^31^ proved that gastrodin protects against glutamate‐induced ferroptosis in HT‐22 cells through Nrf2/HO‐1 signalling pathway. Gai et al^51^ showed that acetaminophen sensitizes erastin‐induced ferroptosis by way of modulation of Nrf2/heme oxygenase‐1 signalling pathway in non‐small‐cell lung cancer. These reports support the hypothesis that Nrf2 suppresses ferroptosis via the Nrf2‐ARE signalling pathway. Consistent with previous reports, we hypothesized that CoNPs induce PIO by promoting osteoblast ferroptosis through the regulation of the Nrf2‐ARE signalling pathway. From our study, we proved that Oltipraz decreased intracellular lipid ROS levels and increased GPX4 activity via the activation of the Nrf2‐ARE signalling pathway.

Currently, treatment strategies for osteolysis induced by wear particles have focused on suppressing inflammation and targeting osteoclasts. However, the therapeutic effects of these methods have proven ineffective and could lead to adverse reactions in other organs. Ohba et al^52^ found that bisphosphonates inhibit osteolysis by inhibiting osteoclasts, but may lead to mandibular lesions, fracture healing disorders, and other adverse reaction. Therefore, there is an urgent need to develop novel strategies for treating PIO. Due to the vital function of osteoblasts in bone metabolism and the pathogenesis of PIO, targeting osteoblasts may be a potential treatment for the disease. All the results discussed above have confirmed the role of ferroptosis in osteoblasts when treated with CoNPs. Meanwhile, CoNPs downregulated Nrf2 to promote the progress of ferroptosis. Currently, in the calvaria resorption model with male C57BL/6J mice, ferroptosis inhibitor Ferrostatin‐1 and Nrf2 activator Oltipraz obviously improved bone microstructure and ameliorated the progression of osteolysis. However, previous research has found that autophagy is also involved in the process of PIO induced by programmed death of osteoblasts, and the systematic dissection of different pathways requires further investigation.

## 5 CONCLUSIONS

**FIGURE 9:**
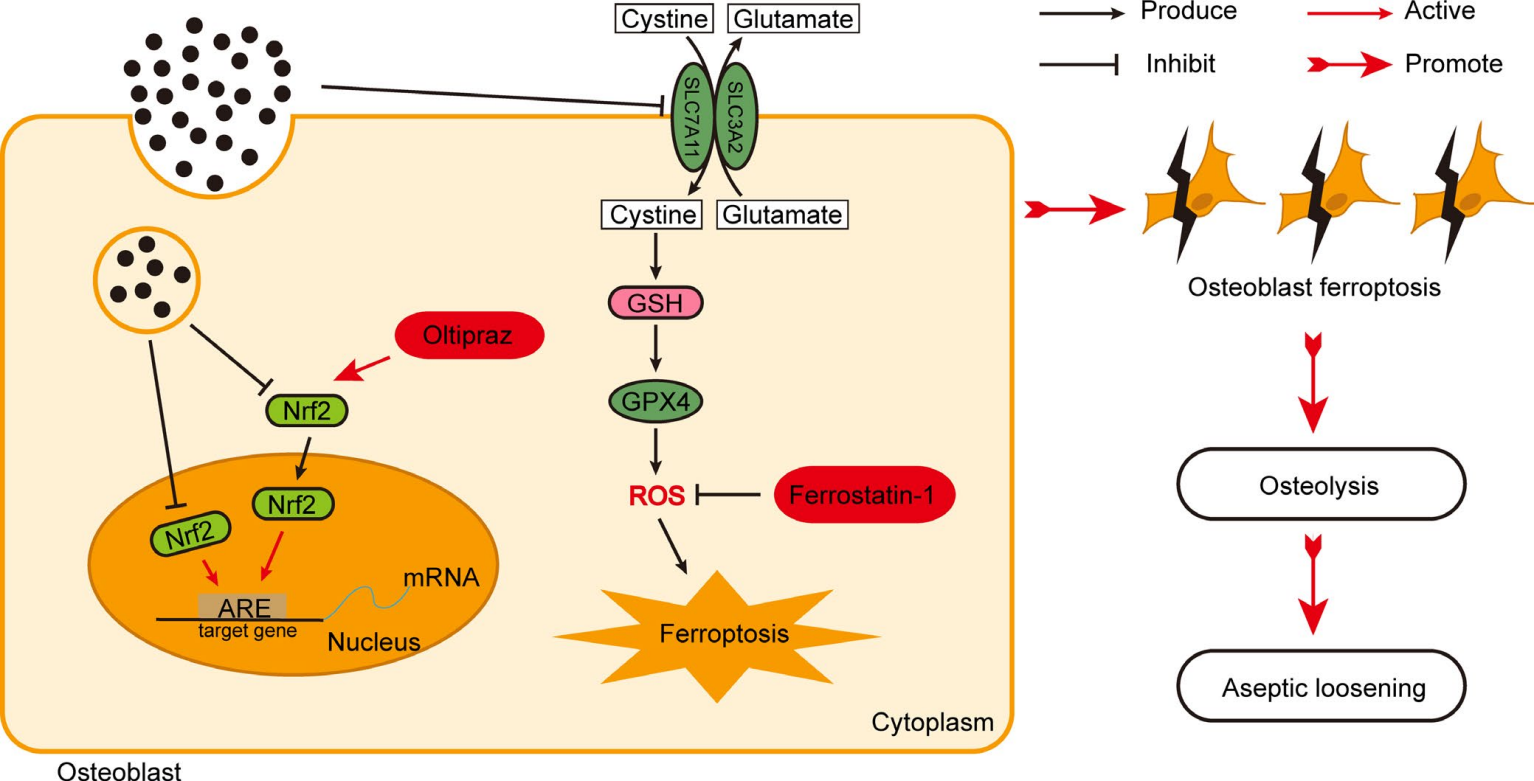
The schematic illustration of CoCrMo‐Nanoparticles induced peri‐implant osteolysis by promoting osteoblast ferroptosis via regulating Nrf2‐ARE signalling pathway

In conclusion, our results highlight that CoCrMo‐Nanoparticles induced peri‐implant osteolysis by promoting osteoblast ferroptosis via regulating Nrf2‐ARE signalling pathway (Figure 9). We carried out RNA‐Seq transcriptome analysis of MC3T3‐E1 osteoblasts, and the results showed that osteoblast ferroptosis could be a vital reason for imbalanced bone metabolism. Marked characterization of ferroptosis was performed in osteoblasts in vitro and in vivo. In addition, all present data clearly show that CoNPs induce PIO by promoting osteoblast ferroptosis through the regulation of the Nrf2‐ARE signalling pathway. More importantly, blocking ferroptosis with Ferrostatin‐1 and Oltipraz significantly ameliorated osteolysis induced by implanted particles. In summary, this study may imply a prospective mechanism underlying wear‐particle‐induced osteolysis and represent a potential therapeutic strategy for treating aseptic loosening.

## CONFLICT OF INTEREST

The authors declare no conflicts of interest for this work.

## AUTHOR CONTRIBUTIONS

Yiming Xu and Weilin Sang contributed to the concept and writing ‐ original draft. Yiming Zhong and Song Xue contributed to data curation and formal analysis. Yiming Xu, Yiming Zhong, Mengkai Yang and Cong Wang contributed to analysis and interpretation of data. Yiming Xu, Weilin Sang, Song Xue, Haiming Lu, Renchun Huan and Xinjie Mao contributed to investigation and methodology. Weilin Sang, Haiming Lu, Libo Zhu and Jinzhong Ma contributed to the visualization, resources and project administration. Chuanglong He and Jinzhong Ma contributed to funding acquisition, conceptualization, supervision and writing ‐ review & editing.

## Supporting information

Appendix S1

## Data Availability

All data included in this study are available upon request from the corresponding author.
