# Supplementary material for: CoCrMo‐Nanoparticles induced peri‐implant osteolysis by promoting osteoblast ferroptosis via regulating Nrf2‐ARE signalling pathway
Source: Cell Prolif. 2021 Oct 11;54(12):e13142. doi: 10.1111/cpr.13142 (PMC8666286; doi:10.1111/cpr.13142)
Supplement: Supplementary file 1 — Appendix S1 [file CPR-54-e13142-s001.docx]

***SUPPLEMENTARY INFORMATION***

**CoCrMo-Nanoparticles induce peri-implant osteolysis by promoting osteoblast ferroptosis via regulating Nrf2-ARE signalling pathway**

Yiming Xu ^1,2^ | Weilin Sang ^1^ | Yiming Zhong ^1,2^ | Song Xue ^1,2^ | Mengkai Yang ^1,2^ | Cong Wang ^1^ | Haiming Lu ^1^ | Renchun Huan ^1^ | Xinjie Mao ^1,2^ | Libo Zhu ^1^ | Chuanglong He ^3^ | Jinzhong Ma ^1^

^1^Department of Orthopedics, Shanghai General Hospital, Shanghai Jiao Tong University School of Medicine, Shanghai, China

^2^Shanghai Bone Tumor Institution, Shanghai, China

^3^State Key Laboratory for Modification of Chemical Fibers and Polymer Materials, College of Chemistry, Chemical Engineering and Biotechnology, Donghua University, Shanghai, China

**Correspondence**

Jinzhong Ma and Libo Zhu, Department of Orthopedics, Shanghai General Hospital, Shanghai Jiao Tong University School of Medicine, Shanghai 200080, China

Emails: [majinzhong1963@sina.com](mailto:majinzhong1963@sina.com)

[libozhu2018@163.com](mailto:libozhu2018@163.com)

Chuanglong He, College of Chemistry, Chemical Engineering and Biotechnology, Donghua University, 2999 North Renmin Road, Shanghai 201620, China

Email: [hcl@dhu.edu.cn](mailto:hcl@dhu.edu.cn)

Yiming Xu, Weilin Sang and Yiming Zhong contributed equally to this work.

**ACKNOWLEDGEMENTS**

This work was financially supported by the National Natural Science Foundation of China (81871795 and 31771048), Shanghai Municipal Health and Family Planning Commission (SHDC12017121), Medical Engineering Cross Research Project of Shanghai Jiaotong University (YG2019QNB37) and Songjiang District Science and Technology Research project (18sjkjgg18).

**METHODS**

**KEGG enrichment analysis**

The MC3T3-E1 cells were stimulated by CoNPs for 24 h, and total RNA was extracted with TRIzol Reagent. Differentially expressed genes were sequenced using the Illumina HiSeq 4000 instrument. For further study, Kyoto Encyclopaedia of Genes and Genomes (KEGG) pathway analyses was performed using OmicStudio tools at <https://www.omicstudio.cn/tool>.

**Western blotting**

We used western blotting to analyse the protein expression. MC3T3-E1 cells were treated with CoNPs (50 μg/ml) in a gradient time (0, 6, 12, 24 and 48 h). Proteins were extracted from MC3T3-E1 cells through ice-cold radioimmunoprecipitation assay lysis buffer after different treatments. Protein concentrations were determined using the BCA protein assay (Beyotime, Shanghai, China). The proteins were resolved using 10% sodium dodecyl sulphate polyacrylamide gel electrophoresis and transferred to a polyvinylidene difluoride membrane. The membranes were blocked with TBS containing 5% skimmed milk, incubated in 0.1% Tween-20 (TBS-T) for 1 h, and incubated with a primary antibody at 4 °C overnight. After incubation with a horseradish peroxidase-conjugated secondary antibody at room temperature for 1 h, membranes were visualised using an enhanced chemiluminescence (ECL) kit. The following primary antibodies were used: SLC7A11 (26864-1-AP, Proteintech), GPX4 (ab125066, Abcam), COX2 (ab62331, Abcam), ACSL4 (ab155282, Abcam), α-tubulin (3873, Cell Signalling Technology). Western blot quantification analysis was performed using ImageJ software (US National Institutes of Health, USA).

**Total RNA isolation and real-time PCR**

MC3T3-E1 cells were treated with CoNPs (50 μg/ml) in a gradient time (0, 6, 12, 24 and 48 h). Total RNA was collected from MC3T3-E1 cells using TRIzol reagent (Thermo Fisher Scientific, Australia) following the manufacturer’s instructions, for different treatments at the indicated time points. PrimeScript RT Master Mix (TaKaRa, Beijing, China) was used to reverse transcribe RNA to cDNA. We used UNICONTM qPCR SYBR Green Master Mix (Yeasen, Shanghai, China) and a LightCycler 96 real-time PCR system (Roche Molecular Systems) to perform real-time PCR. The mRNA expression levels of the target genes were standardised with GAPDH. Primers for the related genes used for real-time PCR are shown in Table S1.

**Cell viability assay**

The effect of different concentration Ferrostatin-1 (0, 0.2, 0.4, 0.6, 0.8, 1, 2 and 4 μM/ml) and Oltipraz (0, 0.1, 1, 2, 5, 10, 20 and 40 μM/ml) on MC3T3-E1 cells were assessed in vitro. MC3T3-E1 cells were cultured in 96-well plates at a density of 6 × 10^3^ cells per well. After corresponding treatments, 10 μl of CCK-8 were added to a 96-well plate and incubated at 37 °C for 2 h. The optical density (OD) at 450 nm was measured by a microplate photometer.

**RESULTS**


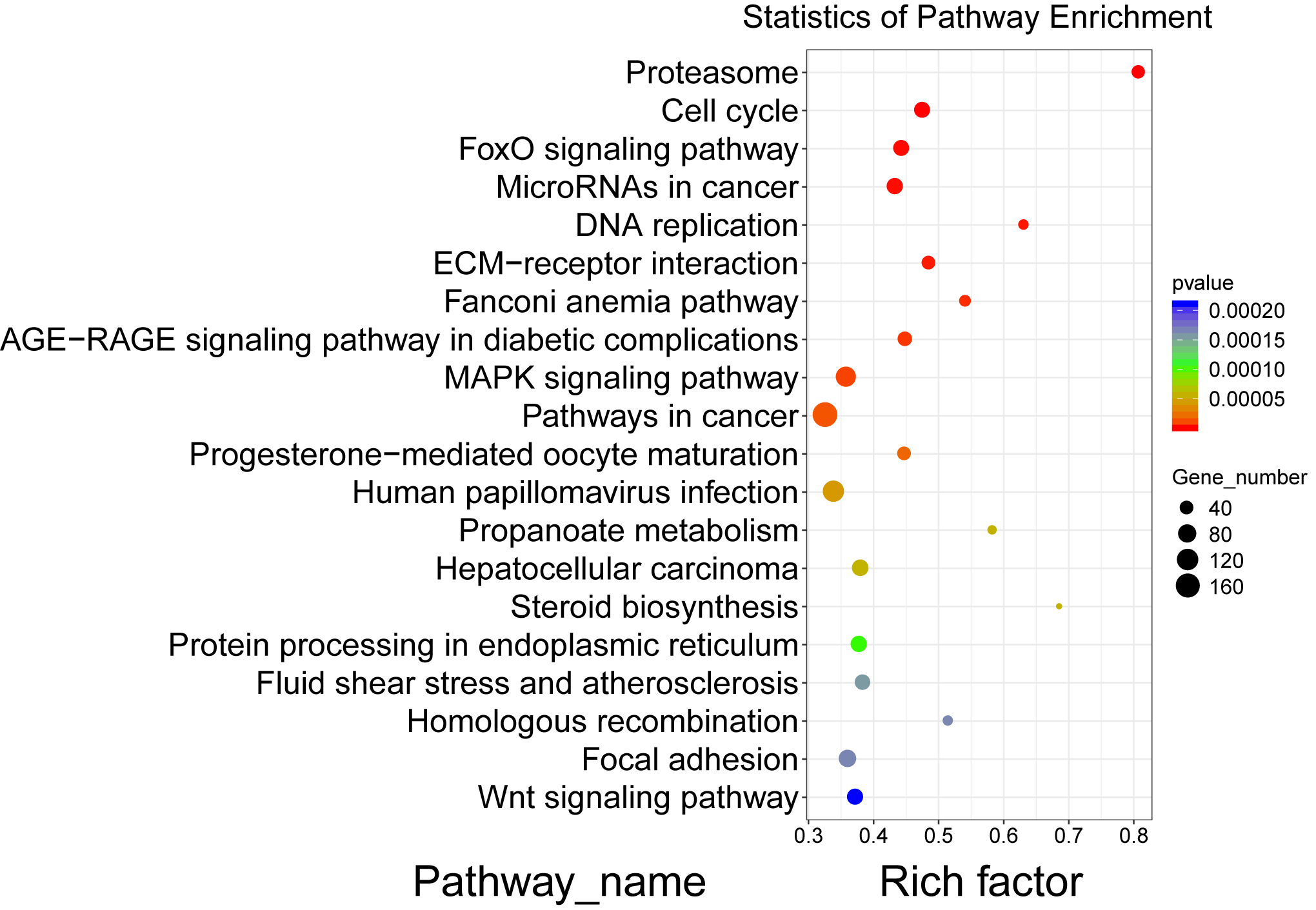


**FIGURE S1** Significant pathway of KEGG Enrichment analysis


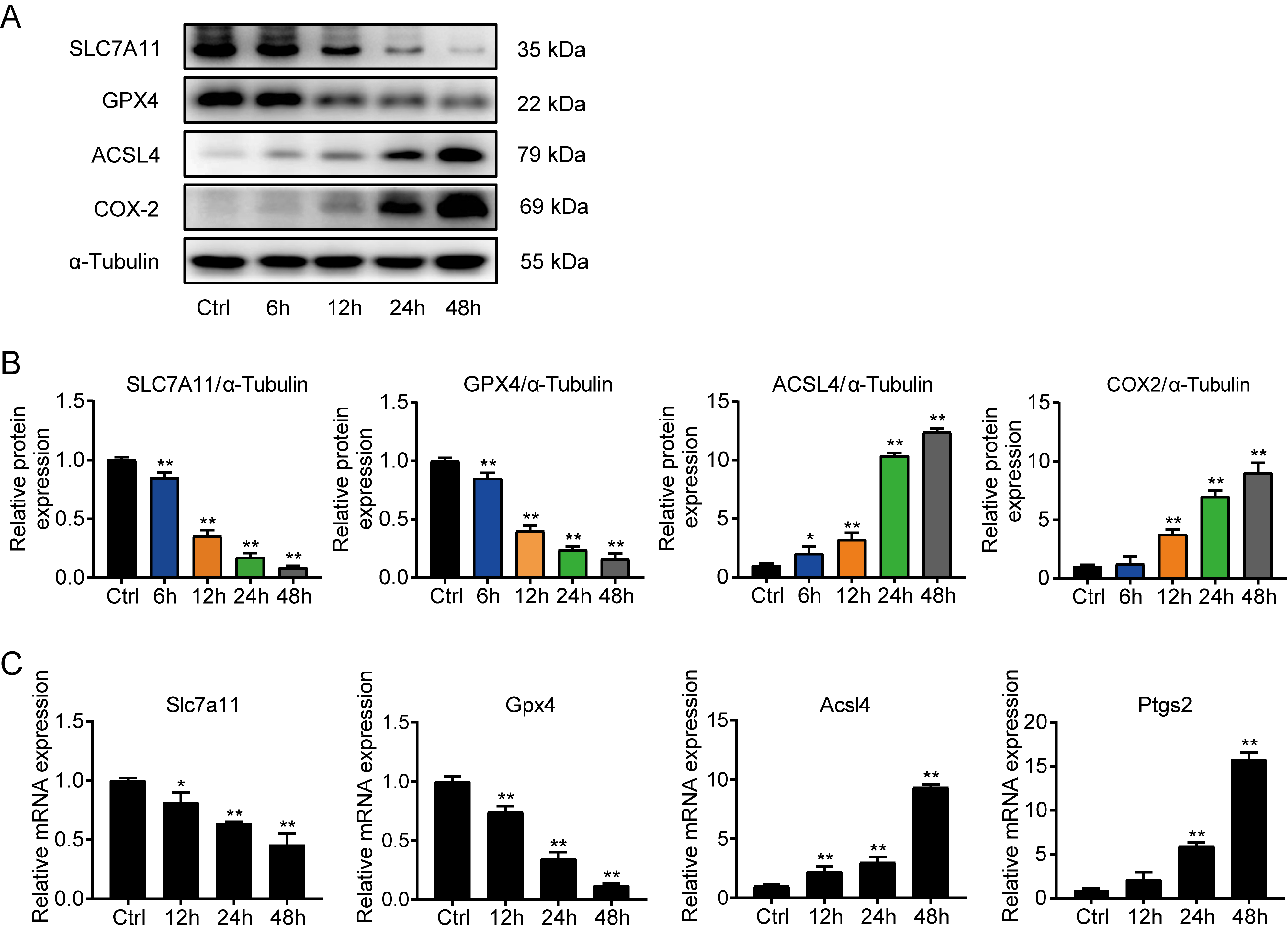


**FIGURE S2** CoNPs induced osteoblasts ferroptosis in vitro. (A) Western blots performed after cells were treated with 50 μg/ml CoNPs for gradient time (0, 6, 12, 24 and 48 h). (B) ImageJ software was used to quantify the density of the western blot bands shown in (A). (C) Relative mRNA expression of Gpx4, Slc7a11, Acsl4, and Ptgs2 in each group. ^*, #^ indicates p < 0.05, ^**, ##^ indicates p < 0.01, ^ns^ indicates not significant.


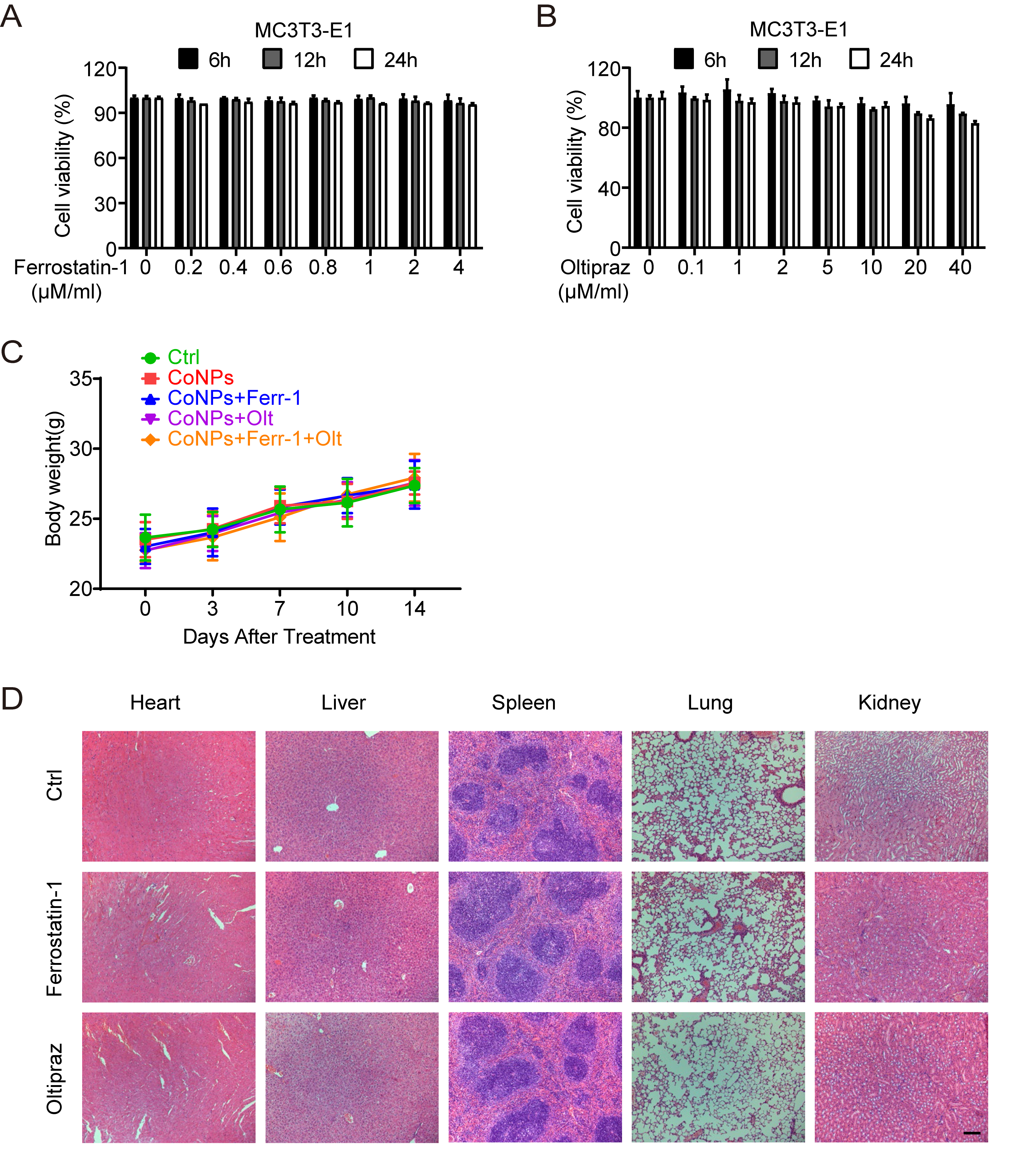


**FIGURE S3** (A) The effect of Ferrostatin-1 on cell viability of MC3T3-E1 cells was measured by CCK-8 assay. (B) The effect of Oltipraz on cell viability of MC3T3-E1 cells was measured by CCK-8 assay. (C) Body weight of rats was recorded after treatment every three days (n = 5). (D) H&E staining of important organs for the Control group, Ferrostatin-1 group and Oltipraz treated group (scale bar: 200 μm).

**TABLE S1** Primer sequences for real-time PCR studies.

| **Gene** | **Primer sequences forward/reverse** |
| --- | --- |
| Runx2 | TGGCTTGGGTTTCAGGTTAG/CCTCCCTTCTCAACCTCTAATG |
| Ocn | GCAATAAGGTAGTGAACAGACTCC/CCATAGATGCGTTTGTAGGCGG |
| β-Catenin | GTTCGCCTTCATTATGGACTGCC/ATAGCACCCTGTTCCCGCAAAG |
| Osterix | GGCTTTTCTGCGGCAAGAGGTT/CGCTGATGTTTGCTCAAGTGGTC |
| Col1a1 | CAATGCTGAAATGTCCCACC/AACAGTCCAAGAACCCCATG |
| Opg | CGGAAACAGAGAAGCCACGCAA/CTGTCCACCAAAACACTCAGCC |
| Gpx4 | CCTCTGCTGCAAGAGCCTCCC/CTTATCCAGGCAGACCATGTGC |
| Slc7a11 | CTTTGTTGCCCTCTCCTGCTTC/CAGAGGAGTGTGCTTGTGGACA |
| Ptgs2 | GCGACATACTCAAGCAGGAGCA/AGTGGTAACCGCTCAGGTGTTG |
| Acsl4 | CCTTTGGCTCATGTGCTGGAAC/GCCATAAGTGTGGGTTTCAGTAC |
| Nfe2l2 | CAGCATAGAGCAGGACATGGAG/GAACAGCGGTAGTATCAGCCAG |
| Keap-1 | TGGGAATAAAGAATGGAGTAGGC/CAAGCAGAGACAATAGACGGG |
| HO-1 | CACTCTGGAGATGACACCTGAG/GTGTTCCTCTGTCAGCATCACC |
